# Justice as care: embedding legal services into HIV care to address health-harming legal needs

**DOI:** 10.1080/09581596.2025.2575803

**Published:** 2025-10-21

**Authors:** Omar Martinez, Sandra Rodriguez, Andrew Maude, Theresa Brabson, Florence Marie Momplaisir, Hervette Nkwihoreze, Robin Davison, Ashley French, Lisa Pozzi, Deja Alvarez, Souhail Malave-Rivera, Amanda Cruz-Gerena, Samantha Morton, Carlos Rodriguez-Diaz, Miguel Muñoz-Laboy

**Affiliations:** a College of Medicine, University of Central Florida, Orlando, FL, USA; b The Philadelphia AIDS Consortium, Philadelphia, PA, USA; c Legal Clinic for the Disabled, Philadelphia, PA, USA; d Perelman School of Medicine, University of Pennsylvania, Philadelphia, PA, USA; e University of Puerto Rico, San Juan, Puerto Rico; f Samantha Morton Consulting LLC, Boston, MA, USA; g Boston University, Boston, MA, USA; h Stony Brook, Stony Brook University School of Social Welfare, Stony Brook, NY, USA

**Keywords:** Health-harming legal needs (HHLNs), Medical-Legal Partnership (MLP), HIV care, legal services, patient-centered interventions

## Abstract

People with HIV face a constellation of social and structural barriers, including unstable housing, discrimination, lack of identification documents, immigration issues, and denial of public benefits, that impede access to and retention in care. These health-harming legal needs are rarely addressed systematically in HIV service settings, despite longstanding calls for structural interventions. This study examines the role and impact of a Medical-Legal Partnership (MLP) embedded within The Philadelphia AIDS Consortium, in collaboration with the Legal Clinic for the Disabled. As part of a larger feasibility and acceptability trial, we conducted follow-up interviews with 95 participants who engaged with the on-site attorney. Recruitment was conducted via venue-based outreach, online ads, and peer referral. Participants reported a substantial reduction in legal needs over time, with more than half reporting no health-harming legal needs (HHLNs) by the 3- and 6-month follow-ups. The most common concerns at baseline, transportation, care planning, and food access, declined significantly. Thematic analysis revealed six key domains of impact: (1) empowerment and knowledge-building; (2) trust, respect, and cultural competence; (3) accessibility and integration of legal services; (4) practical legal resolution; (5) emotional relief and therapeutic benefit; and (6) limitations in legal scope or access. Findings underscore the transformative potential of MLPs in HIV care. When embedded in trusted, culturally responsive settings, legal services can help dismantle structural barriers, foster trust, and support both practical legal resolution and improved mental health outcomes.

## Introduction

People with HIV (PWH) frequently face complex social and structural barriers that extend far beyond clinical care and significantly impact their health outcomes. These barriers often include housing instability, employment discrimination, immigration-related challenges, lack of access to government-issued identification, and the denial or termination of public benefits (Aidala et al., 2016; Bogart et al., 2011; Turan et al., 2017). While these issues are commonly referred to as social determinants of health, they are often deeply legal in nature and fall outside the traditional scope of medical intervention. A growing body of research identifies these issues as health-harming legal needs (HHLNs), civil legal problems that negatively affect a person’s health or access to care. HHLNs occur when individuals are unable to resolve a legal dispute or uphold a legal right, often due to lack of representation, awareness, or access to legal assistance (Bulatovic et al., 2024; Johnson et al., 2024; Morton et al., 2025). These unaddressed legal needs have profound consequences: they are associated with delays in HIV diagnosis and treatment initiation, interruptions in antiretroviral therapy (ART), reduced medication adherence, and poorer clinical outcomes, such as lower CD4 counts and higher viral loads (Aidala et al., 2016; Turan et al., 2017; Morton et al., 2025; Jaén et al., 2024; Tran et al., 2022; Stone et al., 2022; International Nursing Network for HIV/AIDS Research, 2014). In addition, individuals with persistent HHLNs often experience increased psychological stress, miss medical appointments, and demonstrate lower retention in care, all of which are detrimental to sustained viral suppression and the broader goals of ending the HIV epidemic (Morton et al., 2025; Jaén et al., 2024; Patient-Centered HIV Care Model Team, 2020). HHLNs are not peripheral to HIV care, they are central. The consequences of systemic inequities are compounded by underinvestment in civil legal aid and fragmentation between health, public health, and legal sectors. As a result, many people living with HIV encounter legal and social problems that directly interfere with their ability to engage in or remain in care, and yet these problems remain largely unaddressed within the healthcare system (Bradley et al., 2019; CHIWOS Research Team, 2020; Cowden et al., 2025; Dasgupta et al., 2021; Gillot et al., 2022; Karram et al., 2024; Kuhns et al., 2021; Muñoz-Laboy et al., 2022; Sohail et al., 2024; Storholm et al., 2025).

To confront this challenge, the MLP approach provides an evidence-based, equity-centered strategy for integrating legal services into healthcare delivery (Bliss et al., 2013; Griesemer et al., 2023; Kendrick-Allwood et al., 2021; Lively, 2024; Moss et al., 2024; Pettignano et al., 2011; Ryan et al., 2012; Tsai, Jenkins, et al., 2017; Tsai, Middleton, et al., 2017). Rooted in the understanding that many health disparities are driven by unmet civil legal needs, MLPs embed lawyers and legal advocates directly into health care settings to collaborate with providers in identifying and addressing HHLNs (Colvin et al., 2012). This model transforms the clinical encounter by recognizing that social determinants of health are not only risk factors but also points of legal intervention. Through routine screening and cross-sector collaboration, MLPs empower healthcare teams to respond to patients’ legal needs with the same urgency as clinical issues. For example, legal advocates can intervene to prevent eviction, appeal wrongful benefit denials, resolve custody disputes, restore access to health insurance, or secure government-issued identification, all of which are foundational for effective engagement in care and long-term health stability. By removing these barriers, MLPs create the conditions necessary for patients to access treatment, adhere to care plans, and navigate complex health systems. The evidence base for MLPs continues to grow. Studies have shown that MLPs are associated with improvements in housing stability, increased access to SNAP, Medicaid, and disability benefits, reductions in emergency department utilization, and improved patient-reported health and quality of life (Berg et al., 2022; Bulatovic et al., 2024; Gilbert et al., 2019; Johnson et al., 2024; King et al., XXXX; Ngai et al., 2025; Patchen et al., 2023; Statz & Watters, 2024)

When integrated into HIV care settings, MLPs offer a uniquely powerful strategy to address the structural determinants that undermine health outcomes for PWH. Legal interventions, such as securing stable housing, addressing food insecurity, or obtaining government-issued identification, can have a direct and measurable impact on treatment adherence, retention in care, and overall well-being. By resolving these HHLNs, MLPs help to stabilize the social foundations necessary for engagement in HIV care. Importantly, applying a health equity lens (Lewis & Cerezo, 2024; Rosenbaum & Schmucker, 2017) to HHLNs reveals that these challenges are not distributed equally; they are shaped by entrenched systems of structural racism, poverty, and other forms of social exclusion. MLPs not only provide legal remedies but also challenge the broader social conditions that create and perpetuate health disparities. Yet despite a growing evidence base, the integration of legal services into HIV care remains limited, inconsistent, and under-resourced. Legal needs are often screened sporadically, if at all; data systems between health and legal sectors rarely communicate, hindering coordinated care; and funding for preventive legal services remains precarious, with few sustainable reimbursement mechanisms. These systemic gaps represent a missed opportunity to deploy a proven, equity-centered intervention at scale.

The integration of MLPs into HIV care also aligns with a broader movement toward structural competency in medicine and public health. Structural competency encourages providers to recognize how institutions, policies, and systems, rather than individual behaviors alone, produce and reinforce health inequities (Melino et al., 2023; Metzl & Hansen, 2014; Shattuck et al., 2020). By embedding legal advocates within clinical teams, MLPs operationalize structural competency by equipping providers with the tools to act on patients’ structural vulnerabilities. Legal professionals offer not only direct services but also training, consultation, and systems-level advocacy that expand the clinical team’s capacity to respond to social and legal barriers. This interprofessional collaboration enhances both the precision and reach of care delivery, helping organizations identify upstream drivers of poor outcomes and develop more responsive, justice-informed models of care. As HIV care systems seek to meet the goals of the Ending the HIV Epidemic (EHE) initiative, diagnose, treat, prevent, and respond, MLPs offer a practical mechanism to embed structural interventions into service delivery and align clinical care with the realities of patients’ lives.

At the same time, MLPs challenge traditional service boundaries by foregrounding trust, dignity, and care as essential components of both legal and clinical practice. For people with HIV, particularly those from marginalized communities, histories of institutional harm, medical mistrust, and legal system involvement can deter help-seeking. MLPs embedded in trusted, community-based settings, such as HIV/AIDS service organizations, can play a reparative role. When attorneys are co-located in familiar, affirming environments, they are better positioned to build relationships that transcend transactional legal aid. This model emphasizes legal empowerment (Joshi et al., 2022; Pizzamiglio & Kovacs, 2021), not just representation, by supporting clients in navigating systems, advocating for their rights, and building the knowledge and confidence needed to sustain long-term engagement in care. In this way, MLPs reflect an ethics of care approach that centers relational, culturally responsive, and trauma-informed practices. These dimensions are often underexplored in evaluations of structural interventions but are central to the lived experience of clients and the transformative potential of cross-sector collaboration.

This qualitative study examines the role and impact of an MLP embedded within The Philadelphia AIDS Consortium (TPAC), a community-based HIV/AIDS organization in Philadelphia, Pennsylvania. Conducted as part of a broader feasibility and acceptability trial, the study explores the lived experiences of PWH who accessed legal services through the MLP. Through in-depth participant narratives, we illuminate how integrated legal supports influence empowerment, access to care, emotional well-being, and resolution of legal barriers. In doing so, this study contributes critical insights into the transformative potential of MLPs as a structural intervention to promote health equity and improve outcomes for PWH.

## Methods

This qualitative study drew on interviews with participants enrolled at the Medical Legal Partnership site within TPAC, a community-based HIV/AIDS organization in Philadelphia, Pennsylvania, conducted in partnership with the Legal Clinic for the Disabled as part of a larger feasibility and acceptability trial. Participants were initially screened for HHLN by a bilingual (English and Spanish) case manager embedded within the clinical care team. Prior to implementation, all members of the MLP team. including attorneys, case managers, and clinical staff, underwent a comprehensive training program covering civil law, the HIV care continuum, culturally responsive practices, and evidence-informed strategies for identifying and addressing HHLN in clinical settings. Following a positive HHLN screen, the case manager conducted a warm hand-off referral to an on-site attorney, ensuring seamless integration of legal services into care and minimizing barriers to engagement. Legal services provided as part of the MLP included individualized legal representation in areas such as housing stability, public benefits, personal planning (e.g. wills, powers of attorney), and legal name and gender marker changes. In addition to one-on-one legal assistance, the attorney also facilitated ‘Know Your Rights’ workshops open to all patients at the clinic, aimed at increasing legal literacy and self-advocacy. These workshops focused on topics highly relevant to the population served, including housing protections and personal planning. The focus of this qualitative component was to explore the impact of this integrated MLP approach from the perspectives of PWH who engaged with legal services offered on-site.

### Recruitment and sample

Participants were recruited from the Medical Legal Partnership intervention arm of the trial using a multi-pronged strategy designed to reach individuals both in and out of care. Recruitment efforts included venue-based outreach at community events and HIV service locations, targeted online advertisements via social media platforms (e.g. Facebook, Instagram), and peer referrals from individuals already enrolled in the study. Eligible participants were 18 years or older, living with HIV, and engaged in services at the Medical Legal Partnership site. A total of 95 participants who had interacted with the on-site attorney completed follow-up qualitative interviews and were included in this analysis. Participants received a $50 incentive for each follow-up interview completed.

Importantly, participants were not required to enroll in a separate program to access legal services. Instead, the MLP was fully embedded into routine HIV care at the partnering clinic site, the TPAC. Legal service was framed as a standard component of comprehensive, person-centered HIV care. The design and theoretical underpinnings of the MLP were grounded in principles of universal screening, timely and low-barrier access to legal services, and integrated service delivery, aiming to address the structural and legal determinants of health that disproportionately affect people living with HIV.

### Data collection and measures

We conducted descriptive analyses to assess the prevalence and types of HHLNs reported by participants at baseline, 3-month, and 6-month follow-up time points. At each interval, participants were presented with a standardized checklist of potential legal and social concerns and asked: *‘We want to make sure you have all of the help and support you need. If you want help with any of the topics listed below, please let us know by checking them off’.* The checklist included a range of domains relevant to social and legal determinants of health, such as food insecurity, transportation needs, eviction or housing instability, access to health insurance, personal and advanced care planning, and other civil legal issues. The screening tool functioned as a self-reported instrument, designed to capture not only the existence of potential legal needs but also the participant’s expressed desire for assistance. In doing so, it reflects actionable or mobilized needs, indicating both recognition of a concern and readiness to engage with legal services rather than serving as an exhaustive or diagnostic inventory of all legal issues experienced. Findings related to health-harming legal needs and attorney case summaries have been reported in separate publications (Morton et al., 2025).

Qualitative data were collected via structured follow-up interviews conducted by trained study staff. These interviews specifically targeted participants’ experiences with legal services and their perceptions of the impact of the MLP. To capture both the broader and more individualized effects of the intervention, we included two open-ended questions in the follow-up interviews: (1) *‘How do you feel about the interaction with the lawyer?’* and (2) *‘How did your interaction impact you?’* The first question was designed to elicit participants’ reflections on the interpersonal and professional dynamics of their interaction with the on-site attorney. The second question prompted participants to describe the personal significance or perceived outcomes of that interaction, emotionally, practically, or legally. While these questions foregrounded the attorney as a central figure in the delivery of legal services, they also served as an entry point into participants’ broader experience of the MLP model, which integrates legal advocacy into clinical and community-based care settings. This focus reflects our analytic interest in the MLP not only as a structural intervention addressing upstream social and legal determinants of health, but also as an interactional and relational encounter shaped by the approach of the legal provider.

Participants’ responses were recorded in REDCap. Responses were transcribed verbatim, reviewed for accuracy by research staff, and de-identified prior to analysis. The review process included cross-checking audio recordings with transcripts to ensure fidelity to participants’ original statements, followed by the removal or coding of any potentially identifying information to protect participant confidentiality.

### Analytic approach

We employed a rigorous thematic analysis approach to examine participants’ qualitative responses, drawing on established methodologies for identifying, analyzing, and reporting patterns within data (Braun & Clarke, 2021; Nowell et al., 2017). Our analytic process followed a multi-phase, iterative structure to ensure both depth and transparency in interpretation. The first stage involved open coding of transcripts by the first author and two additional members of the research team. Coders independently reviewed the transcripts to generate preliminary codes, which were then compared and discussed in regular debriefing sessions to refine code definitions and resolve discrepancies. This collaborative coding process enhanced the credibility and dependability of our findings by ensuring intercoder reliability and analytic reflexivity. To strengthen rigor, we maintained an audit trail documenting analytic decisions, codebook development, and changes over time. The analysis was both inductive and deductive: inductive in that themes were grounded in participants’ narratives and allowed to emerge organically from the data; and deductive in that our coding was guided by a priori conceptual interests related to the psychosocial and structural impacts of legal services within HIV care. This dual approach enabled us to remain open to unanticipated findings while situating the analysis within the broader theoretical context of structural interventions and health equity.

Codes were organized into higher-order themes through axial coding, which involved identifying relationships among codes and clustering them into meaningful categories. Final themes were developed through consensus among the coding team, with only those themes and illustrative quotes that demonstrated strong intercoder agreement and analytic saturation included in the final framework. Themes were organized around key domains of impact, including empowerment and knowledge-building, trust and respect in provider relationships, accessibility and integration of legal services, legal advocacy and problem resolution, emotional relief and therapeutic value, and structural barriers and limitations in legal scope or access. Representative quotes were selected to exemplify each theme and preserve the richness and authenticity of participants’ voices. These quotations were chosen not only for their relevance but also for their ability to reflect broader patterns across the dataset and convey lived experiences with nuance and clarity.

### Ethical approval

We received ethical approval from Temple University Institutional Review Board (Protocol Number: 28292) and the University of Central Florida Institutional Review Board (STUDY0004712). This study obtained review and approval from the Data and Safety Monitoring Board (DSMB) *for Forging Sustainable Solutions for HIV Continuity of Care through Medical-Legal Partnerships*. The DSMB recommended that the study continue without the need for additional DSMB oversight.

### Informed consent

Informed consent was obtained from all participants, and the study was classified as minimal risk by both the Temple University and University of Central Florida Institutional Review Boards. As such, written consent was not required.

## Results

### Participant characteristics

The sample at the MLP site (n = 95) was demographically diverse and reflected the population most impacted by social and structural barriers to HIV care (Table 1). The average age of participants was approximately 51 years. In terms of gender identity, the sample included a mix of cisgender men and women, with a notable proportion identifying as transwomen and individuals with gender identities not listed in conventional categories. Most participants identified as Black or African American, with a smaller number identifying as White or reporting another race or ethnicity. While most participants were not of Hispanic or Latino/a/x origin, there was notable representation of Puerto Rican individuals. Most participants were single at the time of enrollment, and sexual orientation varied, with participants identifying as straight, gay or lesbian, and bisexual. Nearly all participants reported having some form of health coverage, although a small portion remained uninsured.

### Health-harming legal needs

At baseline, participants reported a high prevalence of health-harming legal needs (HHLN), with the most common concerns including transportation to appointments (49.5%), personal and advanced care planning (46.3%), and access to food resources (40.0%). Emotional or behavioral concerns (25.3%) and social security benefits (16.8%) were also frequently identified. Notably, these needs substantially decreased over time. By the 3-month follow-up, the percentage of participants reporting any HHLN had dropped significantly, with over half (54.8%) reporting no legal needs, a trend sustained at 6 months (53.0%). While certain categories, such as eviction threats and unsafe housing, persisted for a small subset of participants, there was an overall decline in the expressed need for assistance with health-harming legal and social issues over time (Table 2).

### Themes

Six key themes emerged from participant accounts: (1) Empowerment and Knowledge Building—participants reported increased confidence and understanding of their rights; (2) Trust, Respect, and Cultural Competence—participants felt seen and valued, particularly through inclusive and respectful interactions; (3) Accessibility and Integration of Legal Services—embedding legal services within HIV care settings was described as essential and overdue; (4) Legal Advocacy and Practical Resolution—many participants experienced concrete outcomes such as help with benefits, name changes, and legal planning; (5) Relief, Emotional Support, and Therapeutic Value—participants emphasized the emotional and psychological benefits of being heard and supported; and (6) Limitations in Legal Scope or Expertise—some participants faced barriers due to the complexity of their legal needs or challenges accessing legal services on time.

### Empowerment and knowledge building

Many participants described feeling empowered following their legal consultations, highlighting the transformative role that legal service played in helping them better understand their rights and available options across various issues, including housing, immigration, public benefits (e.g. SSI), and identity documentation. This sense of empowerment stemmed not only from the legal advice itself, but also from feeling heard, respected, and supported by the attorney. One participant shared, ‘He educated me about things I didn’t know… how to speak with housing management and what to say and not say’, reflecting a newfound confidence in navigating housing-related challenges. Another remarked, ‘It made me realize that I could get help I needed… empowered me’, emphasizing the psychological and emotional impact of knowing that legal advocacy was available. For many, these encounters marked the first time they had received clear, compassionate guidance tailored to their lived experiences—underscoring the value of embedding legal expertise into healthcare and support services for people with HIV.

### Trust, respect, and cultural competence

Participants frequently emphasized how the lawyer’s approach made them feel respected, seen, and heard—an especially meaningful experience for individuals from historically marginalized communities. These affirming interactions contrasted prior experiences in systems where they may have felt dismissed or misunderstood. One participant shared, ‘I felt very good. The attorney was professional and very friendly. I feel the attorney is good’, capturing a broader sentiment of trust and comfort that many expressed. The use of inclusive language and attention to identity-based needs also resonated strongly. As one participant noted, ‘He was very helpful and personable. He was politically correct with pronouns’, highlighting how simple, respectful gestures can contribute to a more affirming and empowering experience. Another participant who required language support added, ‘[Attorney] was great… we had a translator, and it was really good’, underscoring the importance of cultural and linguistic accessibility in building rapport and delivering effective services. Together, these accounts reveal how legal services, when delivered with inclusivity and cultural humility, can help restore dignity and build trust among individuals who have long navigated exclusionary systems.

### Accessibility and integration of legal services

Many participants emphasized the value of having legal services embedded directly within the same setting as their healthcare or peer navigation services. For individuals who had previously encountered fragmented systems, the presence of a legal professional on-site was described as not only convenient, but essential. Several participants reflected on the barriers they faced when legal services were inaccessible or not offered as part of routine care. As one participant shared, ‘I had 6 case managers and they never told me or referred me to an attorney’, underscoring a longstanding gap in holistic service provision. The integration of legal services was perceived as a significant improvement in care delivery, making it easier to raise issues and receive timely support. Another participant stated, ‘If we have a legal problem, we can consult with the attorney’, expressing a sense of reassurance and confidence in this support system readily available. Others described these interactions as informative and wide-ranging, noting the attorney’s ability to address multiple concerns in one session: ‘[The attorney] answered all my questions and was really helpful. He also informed me about things I did not know about. He shared information about my apartment and landlords. We talked about several topics’. These narratives highlight the importance of co-located legal services in fostering trust, reducing logistical barriers, and enabling proactive problem-solving for people navigating medical and legal systems.

### Legal advocacy and practical resolution

Several participants shared that their legal consultations resulted in concrete, practical resolutions to long-standing issues. These outcomes included assistance with living wills, expungement processes, name changes, credit concerns, and addressing denials of public benefits. The presence of an on-site legal advocate not only clarified confusing processes but also offered a path forward. One participant remarked, ‘He solved the legal issue’, underscoring the tangible relief that timely legal services can provide. Another elaborated on the thoroughness and personal care received: ‘Let me put it this way: he did everything I asked him… every step was done’. Similarly, a participant who needed assistance with advanced planning shared, ‘He was doing a living will for me, he talked to my kids. He was one of the greatest person, every step was done’, highlighting not only the legal guidance but also the compassion and attentiveness that defined the experience. Even when outcomes were still pending, many participants expressed a sense of being on the right path and better equipped to navigate their legal challenges—reflecting the value of accessible legal advocacy embedded within trusted care settings.

### Relief, emotional support, and therapeutic value

Beyond practical outcomes, participants frequently emphasized the emotional relief and sense of validation they experienced through their legal consultations. Being listened to, supported, and taken seriously by a legal professional had a therapeutic effect, particularly for individuals navigating complex or traumatic life circumstances. One participant shared, ‘Made me feel relieved’, while another reflected, ‘It was a warm interaction… very good’, capturing the comfort and human connection that came through the encounter. Even when legal pathways were not immediately clear, the emotional benefit of having someone affirm their experiences and guide them forward was deeply valued. One participant explained, ‘It went well, I got more information on my situation and they gave me thoughts on what I should do’. Expressions like ‘Very satisfied’ were echoed across interviews, underscoring how impactful these interactions were personally. One participant poignantly summed up the therapeutic nature of the support: ‘Let me put it this way: he did everything I asked him. He was doing a living will for me, he talked to my kids. He was one of the greatest person, every step was done’. These accounts highlight how legal partnerships can serve not only as avenues for justice but also as sources of healing and dignity.

### Limitations in legal scope or expertise

While the majority of participants reported positive and impactful experiences, some encounters highlighted the limitations in legal scope or access. In a few cases, the participant’s legal concern extended beyond the lawyer’s expertise or capacity, requiring a referral or additional follow-up. One participant shared, ‘Andrew has not been able to identify a course of action… was going to follow up’, reflecting the ongoing nature of complex legal issues such as Veterans Affairs claims or specialized cases requiring private counsel.

Moreover, a sizable portion of participants indicated they had not yet accessed legal services, whether due to scheduling conflicts, missed follow-ups, or a lack of awareness. Statements such as ‘Haven’t met with lawyer yet’, ‘Did not seek legal services but is going to. Resent the information to him’, and ‘Did not seek legal services yet but will call TPAC next week’, suggest that logistical challenges and delays remain barriers to full engagement.

Even in cases where services were accessed, some legal issues required external referrals, such as to the Mazzoni Center or private attorneys. Still, participants often appreciated the initial support. As one noted, ‘The lawyer was great. We had a translator but it was really good’. These reflections underscore the need for sustained outreach, navigation support, and expanded legal capacity to ensure that all participants—especially those with highly specific or complex legal needs—can receive timely and effective assistance.

## Discussion

This study highlights the profound and multifaceted role that legal services can play in improving the health, stability, and well-being of PWH when delivered through a culturally responsive, co-located model such as the MLP. Findings highlight the critical role that addressing HHLN can play in improving overall well-being and reducing structural barriers to care. The significant reduction in reported HHLN from baseline to the 3- and 6-month follow-ups suggests that the intervention effectively mitigated key social and legal stressors. In particular, the substantial declines in transportation challenges, personal and advanced care planning needs, and food insecurity point to targeted areas where legal and supportive social services can yield immediate benefits. The persistence of some challenges, such as housing instability and unsafe living conditions, underscores the complexity and long-term nature of these problems, which may require more sustained, multisectoral structural responses. While we observed a decrease in the proportion of participants expressing a desire for assistance with HHLNs over the 6-month follow-up period, this trend should be interpreted with caution. The screening tool captures expressed needs that is, legal or social issues that participants both recognized and were willing to seek help for, rather than an objective inventory of all existing legal problems. Therefore, the observed decline more accurately reflects a decrease in the desire for assistance with legal needs, which may suggest that some participants experienced a resolution of prior legal issues, increased capacity to self-manage, or improved trust in available support systems. However, this decline does not necessarily indicate the full absence of unmet legal needs. Overall, these outcomes reinforce the importance of integrating legal services into healthcare and social support systems to address social determinants of health and promote health equity.

The self-reported legal and social needs instrument used in this study was designed to assess not only the existence of potential legal concerns but also whether participants identified those concerns as ones for which they desired assistance. This distinction is important, as the tool captures an expressed or actionable need, reflecting both the recognition of a legal or social issue and a participant’s readiness to engage with support services. As such, it is not intended to serve as a comprehensive or diagnostic inventory of all legal needs a participant may be experiencing. Rather, it offers insight into which issues individuals are willing and able to act upon within the context of their care. We acknowledge that while a desire for assistance can serve as a strong proxy for an unmet legal need, it may not fully reflect the scope, urgency, or severity of the legal harm present. For instance, participants may underreport needs due to stigma, mistrust, uncertainty about legal processes, or a lack of awareness that their situation has a legal remedy. This underscores the need for continued refinement of HHLN measurement tools that are both client-centered and theoretically grounded. Our operationalization of HHLNs is informed by, but conceptually distinct from, traditional civil legal needs surveys, which often seek to catalog the presence or absence of unmet legal issues across populations. In contrast, HHLNs are defined here as socially embedded legal problems that directly or indirectly undermine individual or population health, including issues related to housing, benefits, family law, and personal planning. Capturing the complex interplay between legal vulnerability and health outcomes requires tools that consider not only legal problem prevalence, but also factors such as readiness to seek help, trust in systems, and structural barriers to justice. We view this as an evolving area of inquiry that demands further methodological development and alignment with both public health and legal services research frameworks.

Through thematic analysis of qualitative interviews, we identified key domains of impact, including empowerment, emotional relief, respect and cultural affirmation, and resolution of practical legal concerns related to, for instance, housing, benefits, and identity documentation. Notably, these legal consultations occurred within a trusted community-based HIV service organization, reducing barriers to access and helping to build trust among individuals who have historically been marginalized by health, legal, and social systems. The integration of legal services within an HIV care setting allowed participants to engage with legal professionals who were attuned to their identities, trauma histories, and the daily realities of navigating poverty, discrimination, and systemic exclusion. Participants not only gained critical knowledge of their legal rights and options but also described a renewed sense of confidence, control, and validation, outcomes rarely captured in traditional clinical metrics yet central to long-term health and engagement in care. These findings affirm what many advocates have long asserted: addressing legal needs is not a peripheral issue in HIV care but a core component of equitable, holistic, and effective service delivery (Arnason et al., 2001; Close et al., 2021; Ewais & Banks, 2018; Sage & Warren, 2023).

Furthermore, this study contributes to a growing body of evidence calling for structural interventions that move beyond individual behavior change to address the broader social and legal determinants of health (Agénor et al., 2023; Blankenship et al., 2000; Daly, 2024; Dominick et al., 2016; Olsson et al., 2024; Todić et al., 2020). In a city like Philadelphia, which continues to experience stark racial and economic inequities, high rates of poverty, and one of the highest HIV prevalence rates in the country, the need for such approaches is especially urgent. Embedding legal services in HIV care represents a tangible, scalable, and patient-centered strategy that acknowledges the layered complexity of people’s lives.

However, our findings also reveal ongoing challenges. A notable portion of participants reported not yet accessing legal services, suggesting persistent logistical, informational, or capacity-related barriers. Others encountered legal needs that extended beyond the expertise or scope of the MLP, highlighting the importance of referral networks, interdisciplinary support, and a broader continuum of legal care. Addressing these gaps will require a commitment to expanding legal capacity within community health settings, strengthening partnerships between legal and health sectors, and adopting implementation science frameworks, such as RE-AIM, CFIR, and the Health Equity Implementation Framework (Aerts et al., 2023; Deepa & Judy, 2024; Drake et al., 2021; Finkelstein et al., 2024; Nagy et al., 2025), to guide and evaluate integration efforts.

### Limitations

While this study provides important insights into the value of integrating legal services into HIV care through a MLP approach, several limitations should be acknowledged. First, the data relied on self-reported experiences collected during follow-up interviews, which may be subject to recall bias or social desirability bias. Participants may have overstated the benefits of the legal interaction or underreported challenges due to their relationship with the organization or interviewer. Additionally, the interviews were conducted after a single or limited number of legal interactions, which may not fully capture the long-term impact or effectiveness of the intervention. Second, the sample size was drawn exclusively from TPAC. We acknowledge that the unique context of TPAC, including its long-standing community ties, may have influenced participants’ experiences. Third, not all participants who were referred to legal services successfully engaged with the attorney, and in some cases, issues fell outside the scope or expertise of the MLP attorney, necessitating external referrals. These logistical and capacity-related barriers underscore the importance of further investment in legal infrastructure and coordination across legal networks. Lastly, while the study explored a wide range of legal challenges and impacts, it did not systematically examine differences by key subgroups (e.g. by immigration status, gender identity, or age), which may be important for tailoring future MLP models. Despite these limitations, the study offers a compelling case for integrating legal services into HIV services, and provides valuable qualitative evidence to guide future implementation, scale-up, and evaluation efforts.

## Conclusion

In sum, this study provides compelling evidence that legal services, when integrated into HIV care through a community-centered, culturally responsive MLP model, can empower clients, resolve critical legal concerns, and foster trust and healing. These outcomes are not ancillary but essential to addressing the systemic inequities that continue to drive poor HIV-related health outcomes. As the field of HIV prevention and care seeks innovative and justice-oriented models of care, scaling and sustaining medical-legal partnerships must be part of the broader solution. By meeting people where they are, legally, emotionally, and structurally, we can begin to close the persistent gaps in care and outcomes that continue to harm the most vulnerable among us.

## Figures and Tables

**Table 1.** Demographic characteristics.

	MLP site *n* = 95, 100%
Age (Mean, *SD*)	51.08421 (12.17734)
*Gender expression*	
Cisman	37, 38.9%
Ciswoman	25, 26.3%
Transwoman	17, 17.9%
Cisgender	1, 1.1%
Prefer not to say	1, 1.1%
A gender not listed	14, 14.7%
Genderqueer or Gender nonconforming	0, 0.0%
Transman	0, 0.0%
Non-binary/third gender; Agender	0, 0.0%
*Race*	
Black or African American	77, 81.1%
White	6, 6.3%
Mestizo	0, 0.0%
Native American, American Indian or Alaskan Native	1, 1.1%
Multi-racial	0, 0.0%
Prefer to self-describe	1, 1.1%
Prefer not to say	12, 12.6%
Other native, Indigenous Race	0, 0.0%
Native Hawaiian or Other Pacific Islander	0, 0.0%
Guamanian or Chamorro	0, 0.0%
*Hispanic, Latino/a/x, or of Spanish origin*	
Not Hispanic/Latino/a/x	73, 76.8%
Mexican, Mexican American, Chicano/a/x	5, 5.3%
Puerto Rican	10, 10.5%
Another Hispanic, Latino/a/x or Spanish origin	3, 3.2%
Some other race, ethnicity, or origin	2, 2.1%
Prefer not to say	2, 2.1%
*Marital status*	
Single	71, 74.7%
In a relationship- living together	4, 4.2%
In a relationship- not living together	12,12.6%
Married	4, 4.2%
Other	4, 4.2%
*Sexual orientation*	
Straight	52, 55.3%
Gay or Lesbian	24, 25.5%
Bisexual	13, 13.8%
Queer	0, 0.0%
Asexual	0, 0.0%
Prefer to self-describe	0, 0.0%
Prefer not to say	6, 6.4%
Health coverage (Any)	
Yes	87, 91.6%
No	8, 8.4%

**Table 2.** Health-harming legal needs (HHLN).

HHLN	Baseline	%	3-Months F/U	%	6-Months F/U	%
Food Resources	38	40.0	9	14.5	12	18.2
Utility Bills or Shut-off Notices	15	15.8	8	12.9	9	13.6
Transportation to Appointments	47	49.5	7	11.3	9	13.6
School or Childcare Issues	3	3.2	0	0.0	0	0.0
Health Insurance	6	6.3	0	0.0	2	3.0
Free Tax Preparation	0	0.0	2	3.2	2	3.0
Emotional or Behavioral Concerns (for you or a family member	24	25.3	2	3.2	5	7.6
Safety Issues (for you or a family member	4	4.2	1	1.6	1	1.5
Eviction or Threat of Losing Home	4	4.2	2	3.2	7	10.6
Unsafe Housing or Repairs	10	10.5	5	8.1	7	10.6
Custody	3	3.2	0	0.0	0	0.0
Child Support	0	0.0	0	0.0	0	0.0
Separation or Divorce	0	0.0	1	1.6	0	0.0
Immigration	4	4.2	1	1.6	1	1.5
Social Security Benefits	16	16.8	5	8.1	3	4.5
Employment/Unemployment	7	7.4	2	3.2	3	4.5
Other Benefits (WIC, SNAP, Cash	5	5.3	5	8.1	1	1.5
Personal Planning, Advanced Care Planning (healthcare, financial power of attorney, living will, etc.	44	46.3	5	8.1	6	9.1
Other	5	5.3	10	16.1	10	15.2

## Data Availability

Due to the sensitive nature of the data, which involves people with HIV and their health-harming legal needs, the dataset is not publicly available to protect participant confidentiality.
